# Effect of Soy Isoflavones on Growth of Representative Bacterial Species from the Human Gut

**DOI:** 10.3390/nu9070727

**Published:** 2017-07-08

**Authors:** Lucía Vázquez, Ana Belén Flórez, Lucía Guadamuro, Baltasar Mayo

**Affiliations:** Departamento de Microbiología y Bioquímica, Instituto de Productos Lácteos de Asturias (IPLA), Consejo Superior de Investigaciones Científicas (CSIC), Paseo Río Linares s/n, 33300 Villaviciosa, Spain; lucia.vazquez@ipla.csic.es (L.V.); abflorez@ipla.csic.es (A.B.F.); luciagg@ipla.csic.es (L.G.)

**Keywords:** isoflavones, daidzein, genistein, equol, minimum inhibitory concentration, lactic acid bacteria, bifidobacteria, intestinal bacteria

## Abstract

The present work aimed to assess the susceptibility of dominant and representative bacterial populations from the human gut to isoflavones and their metabolites. To do so, the minimum inhibitory concentration (MIC) of isoflavone glycosides, isoflavone aglycones, and equol to 37 bacterial strains was determined by broth microdilution. Additionally, for 10 representative strains, growth curves, growth rate (μ), and optical density (OD_600 nm_) of the cultures at 24 h were also determined. MICs of daidzin, genistin, daidzein, and genistein were >2048 μg mL^−1^ for all strains assayed, while that of equol ranged from 16 μg mL^−1^ for *Bifidobacterium animalis* subsp. *animalis* to >2048 μg mL^−1^ for Enterobacteriaceae strains. Changes in growth curves, μ, and final OD were observed among the species in the presence of all tested compounds. Genistein reduced μ of *Bacteroides fragilis*, *Lactococcus lactis* subsp. *lactis*, and *Slackia equolifaciens*, while both genistein and equol increased that of *Lactobacillus rhamnosus* and *Faecalibacterium prausnitzii.* Compared to controls, lower final OD in the presence of aglycones and equol were recorded for some strains but were higher for others. Altogether, the results suggest that isoflavone-derived compounds could modify numbers of key bacterial species in the gut, which might be associated with their beneficial properties.

## 1. Introduction

High intakes of soy-containing foods have been epidemiologically associated with less intense menopausal symptoms and a reduced risk of developing cardiovascular and neurodegenerative diseases and cancer [1]. Though soy contains many biologically active substances [2], its beneficial health effects have been attributed to its isoflavone content [3]. Isoflavones are phenolic compounds found naturally in plants (among which soy is one of the richest sources), the chemical structures of which resemble 17-β-oestradiol. They therefore have estrogenic effects [3]. In soy milk and unfermented soy foods, isoflavones mostly appear as isoflavone–glycoside conjugates (daidzin, genistin, glycitin)—the bioavailability and estrogenic activity of which are low [4]. To be absorbed and reach full activity, isoflavone aglycones (daidzein, genistein, glycitein) need to be released from the corresponding glycosides [5]. This is accomplished by cellular β-glucosidases and β-glucosidases from components of the gut microbiota [6]. Isoflavone aglycones can be metabolized further by cellular enzymes, plus others from components of the gut microbiota [7], to produce more active compounds (such as equol from daidzein) or inactive metabolites [8].

Some of the beneficial health effects attributed to isoflavones could come about via the stimulatory or inhibitory modulation of gut microbial populations. However, the effects of isoflavones on gut microbiota have been little examined [9,10,11,12]. Increases in the number of bifidobacteria have been recorded in some studies [10,12], and population sizes within *Clostridium* clusters have been reported to increase in equol producers [10,13]. However, reductions in bifidobacteria and populations of Enterobacteriaceae have been observed in other studies [11]. Such contradictory results may ultimately depend on the baseline size and composition of the bacterial communities in the gut, which can vary widely between subjects [14]. Finally, like many other polyphenols [15], isoflavones and some of their metabolites have been shown to possess a certain antimicrobial activity against bacterial pathogens [16,17,18]. It is thus conceivable that they might directly or indirectly alter the numbers or relative proportions of pivotal bacterial communities for maintaining a healthy microbial balance in the gut.

The present work aimed to examine the possible inhibitory effect of the most common soy isoflavone glycosides (daidzin and genistin), their derived aglycones (daidzein and genistein), and equol, against 37 bacterial strains, including lactic acid bacteria, bifidobacteria, and strains of other dominant and representative bacterial groups in the human gut.

## 2. Materials and Methods

### 2.1. Bacterial Strains, Growth Media, and Culture Conditions

Of the 37 strains used in this study, 25 were type strains of lactic acid bacteria (LAB) and bifidobacterial species obtained from the Laboratory of Microbiology collection in the Belgian Coordinated Collections of Microorganisms (BCCM/LMG) (Ghent University, Ghent, Belgium), 7 were strains (of which 6 were type strains) of species from human intestines obtained from the Deutsche Sammlung von Mikroorganismen und Zellkulturen (DSMZ) (Leibniz Institute, Braunschweig, Germany), and 5 strains of intestinal species were from our own laboratory collection (Table 1). Strains were considered representative of functional bacterial groups within the human gut; they also represent those most commonly used as probiotics. *Lactococci* were grown on M17 agar (Oxoid, Basingstoke, UK) supplemented with 1% glucose (VWR International, Radnor, PA, USA) at 32 °C for 48 h under aerobic conditions. *Streptococcus thermophilus* was cultured on M17 agar (Oxoid) supplemented with 1% lactose (VWR International) at 37 °C for 48 h, under anaerobic conditions. Heterofermentative lactobacilli were recovered on de Man, Rogosa, and Sharpe (MRS) (Merck, Darmstad, Germany) agar plates and incubated for 48 h at 32 °C or 37 °C and under aerobic or anaerobic conditions, depending on the species. Homofermentative lactobacilli and bifidobacteria were grown on MRS agar supplemented with 0.25% l-cysteine (Merck) and incubated at 37 °C for 48 h under anaerobic conditions. Intestinal anaerobic strains (*Bacteroides* spp., *Blautia coccoides Faecalibacterium prausnitzii*, *Ruminococcus obeum*, and *Slackia* spp.) were streaked on Gifu anaerobic medium (GAM) (Nissui, Tokyo, Japan). All strains of these species were incubated at 37 °C for 48 h under anaerobic conditions. Finally, strains of *Escherichia coli*, *Klebsiella pneumoniae*, *Pseudomonas aeruginosa*, and *Serratia marcescens* were grown on brain heart infusion (BHI; Oxoid) agar at 37 °C for 24 h under aerobic conditions.

### 2.2. Determination of Minimum Inhibitory Concentration

The minimum inhibitory concentration (MIC) of the majority of isoflavone glycosides in soy (daidzin and genistin), their respective aglycones (daidzein and genistein), and the isoflavone metabolite equol (all from LC Laboratories, Woburn, MA, USA) were determined using a broth microdilution test, following standard procedures for aerobic [19] and anaerobic bacteria [20] with minor modifications. Briefly, individual colonies from the above plates were suspended in 5 mL of a sterile 0.9% NaCl solution (VWR International) to a McFarland turbidity of 1. The inoculated saline solution was then diluted 1:1000 in the test medium corresponding to the different species (see Table 1) to obtain an approximate final concentration of 3 × 10^5^ cfu mL^−1^. Aliquots (100 μL) of the diluted cell suspensions were poured into microplate wells with 50 µL of two-fold increasing concentrations of the test compounds, ranging from 0.12 to 2048 µg mL^−1^ (the limit of their solubility). MICs were established by visual inspection as the lowest concentration at which no visible growth was observed. All MIC assays were performed in duplicate. Where discrepancies between analyses were observed, a third assay was performed and the mode reported.

### 2.3. Effect of Isoflavone Aglycones and Equol on Bacterial Growth

The growth of bacteria in the presence of daidzein, genistein, and equol was monitored spectrophotometrically, measuring the optical density (OD) throughout culturing. Colonies were collected, suspended in 10 mL of an appropriate liquid medium, and incubated for 24 h under species-specific conditions as stated above. These cultures were then used to inoculate appropriate fresh media (at 1%) supplemented in independent tubes with daidzein, genistein, or equol (all at 32 µg mL^−1^). Cultures to which no phenolic compounds were added were used as controls. Growth was monitored by measuring the OD at 600 nm using the culture medium as a blank. All growth experiments were performed in triplicate; mean results are reported. The bacterial growth rate (μ) was calculated using the formula μ = Ln(N_2_/N_1_)/t_2_−t_1_, where N_1_ was the OD at time 1 (t_1_) and N_2_ was the OD at t_2_. The interval t_1_-t_2_ was selected within the logarithmic growth phase of the different species and strains.

### 2.4. Statistical Analysis

Statistical analysis of the data was performed using the 3.2.5. version of the free R software (The R Foundation, Boston, MA, USA). Normality of the data was checked by the Shapiro–Wilk test. Mean differences between control cultures and cultures with isoflavones were assessed using the Student’s *t*-test.

## 3. Results and Discussion

All strains grew at the maximum concentration of isoflavone glycosides (daidzin and genistin) and isoflavone aglycones (daidzein and genistein) used (MICs > 2048 µg mL^−1^). In contrast, susceptibility to equol ranged widely, from 16 µg mL^−1^ to 2048 µg mL^−1^ (Table 1). The strain most susceptible to equol was *B. animalis* subsp. *animalis* (MIC = 16 µg mL^−1^), while the most resistant strains belonged to the Gram-negative species *E. coli* and *K. pneumoniae* (MIC = 2048 µg mL^−1^). Eight strains, among which five species of lactobacilli, *Slackia isoflavoniconvertens*, and *P. aeruginosa* were found, showed an MIC of 1024 µg mL^−1^. The tested strains of *Lactobacillus delbrueckii* subsp. *bulgaricus*, *Bacteroides fragilis*, *Bacteroides thetaiotaomicron*, and *Slackia equolifaciens* showed moderate susceptibility to equol (MIC = 64 µg mL^−1^ for all).

Overall, these results agree well with those reported in the literature, in which isoflavones lacking prenyl and hydroxyl groups at certain positions of the isoflavone ring structure—such as daidzin, genistin, daidzein and genistein—have shown no major antimicrobial activity [17]. Other studies describing isoflavones to have low antibacterial activity against Gram-negative bacteria have also been reported [16,17,18]. However, all these works had the aim of assessing isoflavones and their faecal-derived metabolites as potential antibacterial agents for counteracting the rise of antibiotic resistance among pathogens; this is why pathogenic species have been analysed so far [17]. In this work, a majority of strains under analysis were shown not to be inhibited by the tested compounds at concentrations higher than those reached at a physiological level (~200 µg mL^−1^ of intestinal content under usual treatment regimens; [11]). However, due to the large microbial complexity and diversity within the human gut [14,21], the response to isoflavones and their metabolites of members of bacterial groups others than those analysed in this study might be different.

**Figure 1:**
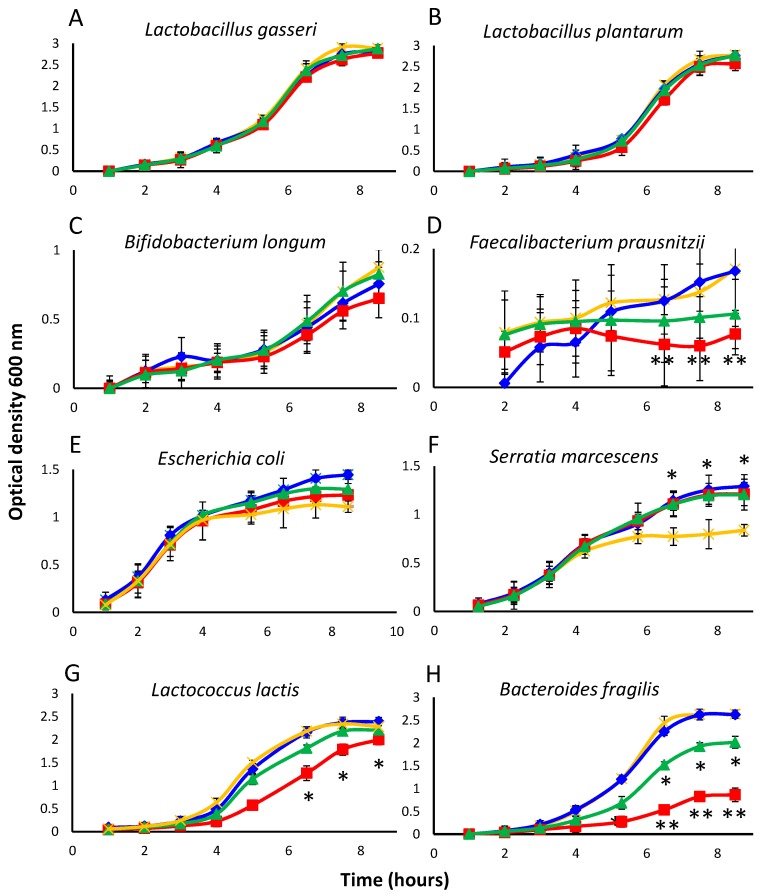
Growth curves of representative strains (**A**–**H**) as an average of the optical density (OD) measures of triplicate cultures in the presence of the soy isoflavone aglycones daidzein (in blue) and genistein (in red), and the daidzein-derived metabolite equol (in green) (all at 32 μg mL^−1^), as compared to a control without additives (in orange). Note that OD scale is different for different species. Mean values were compared by the Student’s *t*-test. Vertical bars show standard deviations (SD). Statistical significance: * *p* ≤ 0.05, ** *p* ≤ 0.01.

To determine whether isoflavones and equol could affect bacterial growth despite their high MIC values, the growth curves of 10 strains belonging to representative groups were investigated under specific culture conditions (see Section 2.1 and Table 1). As MICs of the isoflavone glycosides and isoflavone aglycones resulted identical, the former compounds were not tested in this assay. Since complete inhibition was not intended, the compounds to be assayed were added at a concentration below their MIC values (32 μg mL^−1^). Controls were prepared in which no phenolic compound was provided. Cultures were sampled hourly for the first 8 h of incubation and also after 24 h (at which time the maximum population size was attained). As expected, the growth kinetics recorded varied widely between bacterial groups (Figure 1). Except for slow-growing species (*Sl. equolifaciens*, *Sl. flavoniconvertens*, *Faecalibacterium prausnitzii*), standard deviation between assays was rather low for a microbial test, ranging from 0.03 to 0.23. *Sl. equolifaciens* did not appreciably grow during the first 8 h of incubation in any of the cultures. Broadly speaking, growth of the majority of the strains during these first 8 h (up to the beginning of the stationary phase in most cases) was very similar in the presence or absence of the test compounds, suggesting them to have no effect. Such was the case for *Lactobacillus gasseri*, *Lactobacillus plantarum*, *Bifidobacterium longum, E. coli*, and *S. marcescens* (Figure 1A–C,E,F, respectively). Growth curves similar to those of *L. gasseri* and *L. plantarum* were also obtained for *Lactobacillus rhamnosus* (data not shown). In contrast, cultures of *F. prausnitzii*, *Lactococcus lactis* subsp. *lactis*, and *Bact. fragilis* were inhibited by equol and even strongly by genistein (Figure 1D,H,G, respectively). *E. coli* and *S. marcescens* grew better in the presence of isoflavones and equol than in the control cultures, although the difference was statistically significant for *S. marcescens* only (Figure 1F).

Deconjugation of isoflavone glycosides leads to the release of free glucose [6], which could then be used as a fuel. However, degradation of aglycones by certain species and their use as an energy source cannot be discarded. Indeed, beyond the conversion of daidzein into equol and genistein into 5-hydroxyequol, the catabolic profiling of soy aglycones and their derived metabolites by (intestinal) bacteria has scarcely been addressed [22,23].

**Figure 2:**
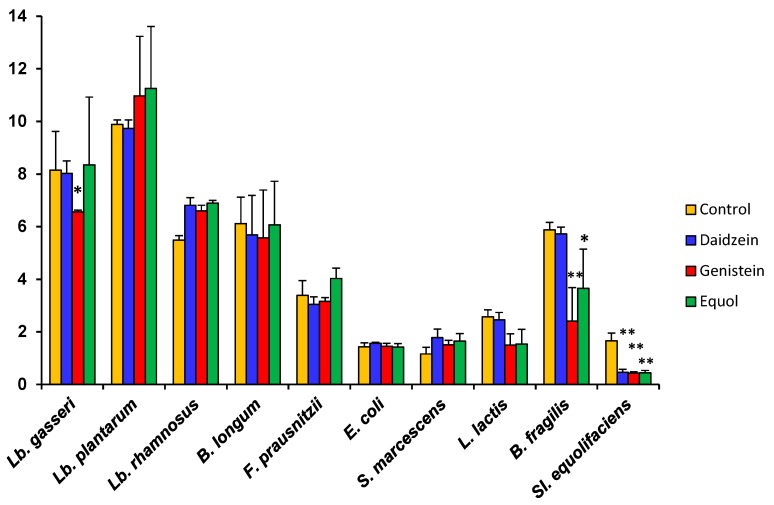
Final optical density (OD) at 600 nm after 24 h incubation of ten bacterial strains in the presence of 32 μg mL^−1^ of either daidzein, genistein, or equol, as compared to a control without phenolics. Standard deviation (SD) is indicated by vertical bars. Mean values were compared by the Student’s *t*-test. Statistical significance: * *p* ≤ 0.05, ** *p* ≤ 0.01.

As compared to the controls, daidzein causes small increases or decreases in the growth rate (μ) depending on the species (Table 2). μ also decreased moderately in some strains when either genistein or equol was present in the culture medium, but increases were scored for some others. In accordance with the shape of their growth curves, the decrease in µ was particularly high for *Bact. fragilis*, *L. lactis* subsp. *lactis*, and *Sl. equolifaciens*. The enhanced growth rate of *Lb. rhamnosus* and *S. marcescens* in the presence of genistein and equol (Table 2) strongly suggests that somehow these species can degrade and use these compounds as an energy source. The catabolism of isoflavone glycosides, aglycones, and equol by strains of these species is currently underway. A particular case was *F. prausnitzii*. Equol and genistein inhibited growth of this species during the first 8 h of culture, but they both increased its µ value (calculated for this strain between 20 and 24 h). To examine the effects of aglycones and equol on the maximum optical density (OD) attained by cultures, this parameter was evaluated for the 10 selected strains at 24 h (Figure 2). Compared to the controls and reinforcing the observed changes in the μ, the presence of isoflavone aglycones or equol led to a lower final OD for some strains but higher for others. Of these changes, statistical significance was only found for the inhibition of *Lb. gasseri* by genistein, *Bact. fragilis* by both genistein and equol, and *Sl. equolifaciens* by all tested compounds.

The inhibitory activity of genistein against pathogens such as *Staphylococcus aureus* has been repeatedly reported [18,24]. As anticipated above, though the chemical structure of daidzein and genistein are very similar (except for the absence of an OH group in daidzein at position 5) [25], genistein inhibits DNA topoisomerease IV while daidzein does not [17], perhaps explaining its stronger antimicrobial action. It was surprising that the growth of *Sl. equolifaciens* was severely inhibited by all the test compounds; this and *Sl. isoflavoniconvertens* were the only equol-producing organisms among the tested bacteria. The equol used in this study was a racemic mixture of *R*- and *S*-enantiomers, while only the latter is produced endogenously in the gut [8]. Therefore, as for some physiological effects [26], the antimicrobial action of the native equol might differ from that reported here. Moreover, soy isoflavones are metabolized into a vast array of chemically-related phenolic compounds [27,28] such as dihydrodaidzein, dihydrogenistein, tetrahydrodaidzein, *O*-desmethylangolensin (*O*-DMA), 5-hydroxyequol, and others [7,8,22], whose antimicrobial behaviour was not tested in this study. In addition, other phenolics, such as 4-ethylcatechol, 3-phenylpropionic acid, 3-hydroxyphenylacetic acid, and 4-hydroxy-5-phenylvaleric acid, have also been recorded to increase their faecal concentrations after isoflavone consumption [22]. Intermediate or end-product metabolites might have a range of biological properties, including antimicrobial activity. In fact, an antimicrobial effect of phenylacetic and phenylpropionic acids has already been reported, particularly against Gram-negative intestinal pathogens [29]. To provide a complete picture of how these compounds affect communities of gut bacteria, the antimicrobial properties of more isoflavone-derived phenolic compounds against representative gut bacteria should be examined. Besides, the use of culture-independent molecular methods to assess the quantification of bacterial growth (such as real-time quantitative PCR) could bring about more accurate results than those obtained by the culturing approach used in this work.

## 4. Conclusions

In conclusion, soy isoflavones and their metabolites are thought to have a range of beneficial health effects, which might be exerted through the modulation of bacterial populations in the human gut [1,3,30]. However, except for a few pathogens, studies examining the effects of these phenolic compounds on bacterial growth and metabolism have yet to be reported. To our knowledge, this is the first paper to report the resistance/susceptibility profiles of members of the commensal and beneficial bacterial communities of the human gut to isoflavones. The related parameters MIC, growth rate, and final growth estimate the competitiveness and fitness of bacteria in the presence of the compounds under study. Since isoflavone aglycones and equol can modify one or more of the variables examined, it might be concluded that when consumed either in food or in supplements, they may modify the total numbers and/or their relative proportions of specific bacterial communities in the gut. These modulatory effects on the intestinal bacterial populations might be associated with the beneficial properties attributed to soy consumption.

## Figures and Tables

**Table 1:** Bacterial strains, assay conditions for the minimum inhibitory concentration (MIC), and MIC results of equol to the intestinal species and strains under study.

Bacterial Strains		MIC Assay Conditions			MIC Results
Bacterial Group/Species	Strain Code	Medium	Temperature	Atmosphere	Equol (μg mL^−1^)
Lactic acid bacteria					
*Lactococcus* (*Lc.*) *lactis* subsp. *cremoris*	LMG 6987^T^	IST ^a^	32 °C	Aerobiosis	256
*Lc. lactis* subsp. *lactis*	LMG 6890^T^	IST	32 °C	Aerobiosis	128
*Streptococcus termophilus*	LMG 6896^T^	IST-Lac ^b^	37 °C	Anaerobiosis	256
*Lactobacillus* (*Lb.*) *brevis*	LMG 6906^T^	LSM ^c^	32 °C	Aerobiosis	256
*Lb. casei*	LMG 6904^T^	LSM	32 °C	Aerobiosis	1024
*Lb. fermentum*	LMG 6902^T^	LSM	37 °C	Aerobiosis	1024
*Lb. paracasei* subsp. *paracasei*	LMG 13087^T^	LSM	32 °C	Aerobiosis	1024
*Lb. pentosus*	LMG 10755^T^	LSM	32 °C	Aerobiosis	1024
*Lb. plantarum*	LMG 6907^T^	LSM	32 °C	Aerobiosis	1024
*Lb. reuteri*	LMG 9213^T^	LSM	37 °C	Aerobiosis	512
*Lb. rhamnosus*	LMG 6400^T^	LSM	37 °C	Aerobiosis	512
*Lb. sakei* subsp. *sakei*	LMG 9468^T^	LSM	32 °C	Aerobiosis	256
*Lb. acidophilus*	LMG 9433^T^	LSM-Cys ^d^	37 °C	Anaerobiosis	512
*Lb. delbrueckii* subsp. *bulgaricus*	LMG 6901^T^	LSM-Cys	37 °C	Anaerobiosis	64
*Lb. delbrueckii* subsp. *delbrueckii*	LMG 6412^T^	LSM-Cys	37 °C	Anaerobiosis	256
*Lb. delbrueckii* subsp. *lactis*	LMG 7942^T^	LSM-Cys	37 °C	Anaerobiosis	128
*Lb. gasseri*	LMG 9203^T^	LSM-Cys	37 °C	Anaerobiosis	128
*Lb. helveticus*	LMG 6413^T^	LSM-Cys	37 °C	Anaerobiosis	1024
*Lb. johnsonii*	LMG 9436^T^	LSM-Cys	37 °C	Anaerobiosis	512
Bifidobacteria					
*Bifidobacterium* (*B*.) *adolescentis*	LMG10502^T^	LSM-Cys	37 °C	Anaerobiosis	256
*B. animalis* subsp. *animalis*	LMG 10508^T^	LSM-Cys	37 °C	Anaerobiosis	16
*B. animalis* subsp. *lactis*	E43	LSM-Cys	37 °C	Anaerobiosis	128
*B. breve*	LMG 13208^T^	LSM-Cys	37 °C	Anaerobiosis	256
*B. longum* subsp. *longum*	LMG 13197^T^	LSM-Cys	37 °C	Anaerobiosis	256
*B. pseudolongum* subsp. *pseudolongum*	LMG 11571^T^	LSM-Cys	37 °C	Anaerobiosis	128
*B. termophilum*	LMG 21813^T^	LSM-Cys	37 °C	Anaerobiosis	256
Other intestinal bacteria					
*Bacteroides (Bact*.) *fragilis*	DSM 2151^T^	M1 ^e^	37 °C	Anaerobiosis	64
*Bact. thetaiotaomicron*	DSM 2079^T^	M1	37 °C	Anaerobiosis	64
*Blautia coccoides*	DSM 935^T^	M1	37 °C	Anaerobiosis	256
*Faecalibacterium prausnitzii*	DSM 17677	M1	37 °C	Anaerobiosis	256
*Ruminococcus obeum*	DSM 25238^T^	M1	37 °C	Anaerobiosis	256
*Slackia* (*Sl*.) *equolifaciens*	DSM 24851^T^	M1	37 °C	Anaerobiosis	64
*Sl. isoflavoniconvertens*	DSM 22006^T^	M1	37 °C	Anaerobiosis	1024
*Escherichia coli*	E-73	IST	37 °C	Aerobiosis	2048
*Klebsiella pneumoniae*	K-78	IST	37 °C	Aerobiosis	2048
*Pseudomonas aeruginosa*	PS-25	IST	37 °C	Aerobiosis	1024
*Serratia marcescens*	S-54	IST	37 °C	Aerobiosis	512

^a^ IST, IsoSensitest (Oxoid); ^b^ IST-Lac (IST + 1% lactose); ^c^ LSM, Lactic acid bacterium susceptibility test medium (90% IST + 10% de Man, Rogosa and Sharpe (MRS)); ^d^ LSM-Cys (LSM + 0.03% cysteine); ^e^ M1 (90% IST + 10% Gifu Anaerobic Medium (GAM) + 0.25% cysteine). MICs were assayed in duplicate or triplicate; when discrepancies were found, the mode was reported.

**Table 2:** Growth rate of selected bacterial strains in cultures supplemented with daidzein, genistein, or equol at a final concentration of 32 µg mL^−1^ as compared to that in control cultures without isoflavone phenolics.

Strain/Culture Conditions	Growth Rate ^a^ (µ) h^−1^	Species/Culture Conditions	Growth Rate (µ) h^−1^
*Lb. gasseri* LMG 9203 ^b^		*Bact. fragilis* DSM 2151 ^d^	
Control	0.747	Control	0.681
Daidzein	0.739	Daidzein	0.701
Genistein	0.738	Genistein	0.153
Equol	0.738	Equol	0.591
*Lb. plantarum* LMG 6907 ^b^		*E. coli* E-73 ^e^	
Control	0.791	Control	0.772
Daidzein	0.755	Daidzein	0.775
Genistein	0.776	Genistein	0.820
Equol	0.722	Equol	0.787
*Lb. rhamnosus* LMG 6400 ^b^		*S. marcescens* S-54 ^e^	
Control	0.867	Control	0.808
Daidzein	0.704	Daidzein	0.792
Genistein	0.942	Genistein	0.874
Equol	0.962	Equol	0.838
*L. lactis* subsp. *lactis* LMG 6890 ^c^		*Sl. equolifaciens* DSM 24851^T^	
Control	0.965	Control	0.238
Daidzein	0.736	Daidzein	0.174
Genistein	0.595	Genistein	0.121
Equol	0.799	Equol	0.153
*B. longum* subsp. *longum* LMG 13197 ^b^		*F. prausnitzii* DSM 17677	
Control	0.581	Control	0.187
Daidzein	0.472	Daidzein	0.181
Genistein	0.516	Genistein	0.232
Equol	0.581	Equol	0.227

^a^ The specific growth rate (μ) under the culture conditions was calculated as μ = Ln(N_2_/N_1_)/t_2_−t_1_, where N_1_ was the OD at t_1_ and N_2_ was the OD at t_2_. To calculate µ, a representative t_1_-t_2_ interval within the logarithmic growth phase of the cultures was selected. ^b^ de Man Rogosa and Sharpe (MRS) broth supplemented with 0.25% cysteine. ^c^ M17 broth supplemented with 1% glucose. ^d^ Gifu Anaerobic Medium (GAM) broth supplemented with 0.5% arginine. ^e^ Luria-Bertani (LB) broth.
